# Symptoms that may be stress-related and lead to exhaustion disorder: a retrospective medical chart review in Swedish primary care

**DOI:** 10.1186/s12875-018-0858-7

**Published:** 2018-10-30

**Authors:** Annika Adamsson, Susanne Bernhardsson

**Affiliations:** 1 Hovås Askim Familjeläkare och BVC, Askims Torg 5, 436 43 Askim, Sweden; 2 Närhälsan Research and Development Primary Health Care, Region Västra Götaland, Kungsgatan 12, 6th floor, 411 18 Gothenburg, Sweden; 3 Department of Health and Rehabilitation, The Sahlgrenska Academy, Institute of Neuroscience and Physiology, University of Gothenburg, Gothenburg, Sweden

**Keywords:** Stress, Exhaustion disorder, Burnout, Primary health care, General practice, Stress related mental health problems, Early detection

## Abstract

**Background:**

Mental illness, and particularly stress-related disorders such as exhaustion disorder, is continuously increasing in today’s society. It is important to identify patients who consult for potentially stress-related symptoms early, before the stress condition develops into an exhaustion disorder. The purpose of the study was to investigate the frequency of different presenting complaints for which patients had consulted in the two years preceding receipt of their exhaustion disorder diagnosis, and to explore potential associations between stress-related presenting complaints and demographic factors, as well as comorbidity and other potentially stress-inducing factors.

**Methods:**

This was a retrospective medical chart review of presenting complaints of adult patients with exhaustion disorder two years preceding receipt of diagnosis at a primary healthcare centre in western Sweden.

**Results:**

Exhaustion disorder was diagnosed in 126 patients at the healthcare centre during the study period. Charts were available for 115 patients (76% women, mean age 47 years). Charts were reviewed with regard to presenting complaints, demographic data and comorbidity. Average number of general practitioner visits during the two years preceding the diagnosis was 5.2 (SD 3.7). The two most common complaints were infection and anxiety/depression, presented by 49% and 46%, respectively. Other stress-related complaints seen to in more than 30% of the patients were stress, other pain, fatigue, gastrointestinal symptoms, and sleep disturbances. Back pain and fatigue were more frequent in patients over 40 years. A majority of the patients also had mental (53%) or somatic (61%) comorbidity. Comorbidity was more frequent in older patients. No significant gender differences were found.

**Conclusions:**

Patients with exhaustion disorder appear to consult their general practitioner numerous times with stress-related complaints in the years preceding their diagnosis. The findings indicate which presenting complaints general practitioners may need to be more attentive to so that patients at risk of developing exhaustion disorder can be identified earlier and get the support they need. Addressing stress factors earlier in the course of illness and preventing the development of exhaustion disorder may contribute to a reduced burden for both individual patients and for society, with a reduction in sick leave and societal costs for mental illness.

## Background

Exhaustion disorder was introduced as a medical diagnosis in Sweden by the Swedish National Board of Health and Welfare in 2010. The proposal for this new diagnosis originated during a study of persons on long-term sick leave due to a mental diagnosis and where workplace conditions were considered to play an important role [1]. It was considered important to define specific diagnostic criteria for stress-related exhaustion, because the terms ‘burnout’, ‘exhaustion’ and ‘stress’ were not defined clearly and were not uniformly used. Exhaustion disorder has been proposed as the most valid clinical equivalent of burnout [2]. Although conceptually related and sometimes used synonymously, burnout is an unspecific term, with various definitions and of psychological origin, whereas exhaustion disorder is a more specific term and a clearly defined clinical diagnosis. The diagnostic criteria include physical and mental exhaustion, cognitive dysfunction, sleep disturbance, and physical symptoms [1]. They are presented in their entirety in Table 1.

**Table 1:** Diagnostic criteria for exhaustion disorder (Swedish National Board of Health and Welfare)

Diagnostic criteria for exhaustion disorder, ICD-10 code F 43.8A	
A. Physical and mental symptoms of exhaustion during a minimum of two weeks. The symptoms have developed in response to one or more identifiable stressors which have been present for at least six months.	
B. Markedly lack of mental energy, which is manifested by reduced initiative, reduced endurance, or prolonged recovery time after mental strain.	
C. At least four of the following symptoms have been present most days during the same two-week period: 1) Concentration difficulties or memory problems 2) Markedly reduced ability to manage demands or to perform under time pressure 3) Emotional instability or irritability 4) Sleep disturbances 5) Marked physical weakness or fatigue 6) Physical symptoms such as pain, chest pain, palpitations, gastrointestinal symptoms, dizziness, or sensitivity to sound	
D. The symptoms cause clinically significant suffering or reduced ability to function at work, socially, or in other important situations.	
E. The symptoms are not related to direct physiological effects of a substance (e.g. drug abuse, medication) or somatic disease/injury (e.g. hypothyroidism, diabetes, infectious disease).	
F. If the criteria for major depressive disorder, dysthymic disorder or generalized anxiety disorder concurrently are fulfilled, exhaustion disorder should be used as a secondary diagnosis.	

Exhaustion symptoms are developed as a consequence of exposure to one or several identifiable stress factors that have existed for a minimum of six months [1]. Symptoms are dominated by a substantial lack of mental energy, manifesting itself as lack of initiative, reduced stamina, or prolonged recovery after mental strain [1]. Work-related symptoms experienced by patients have recently been described as a sense of ‘work dissonance’, disrupted workflow, feeling out-of-sync, and social isolation [3].

The main cause of exhaustion disorder is believed to be stress; predominantly work-related but sometimes due to personal life circumstances [1]. Stress has been defined as an imbalance between demands placed on us and our ability to manage them [4]. Stress factors can cause acute or post-traumatic stress disorder while long exposure to stress without sufficient opportunity for recovery may lead to exhaustion disorder [5]. In classic stress theory, a general adaptation syndrome is described, in which stress reactions are divided into three phases [6]. After an initial alarm reaction and a state of resignation, the third phase entails a state of somatic and mental exhaustion that can cause joint inflammations, cardiovascular disease, gastrointestinal disease, type 2 diabetes, and mental illness [6, 7].

Mental illness—of which exhaustion disorder is one of the most common diagnoses—has become the primary cause of sickness absence, in Sweden as well as in other countries [5, 8]. After a period of stagnating sick leave statistics in the early 2000s, sick leave due to mental disorders began to rise again in 2010, and is today the most common reason for new sick leave registrations [4, 9]. From 2011 to 2016, the number of individuals on sick leave for mental disorders more than doubled, from 42,000 to 89,000, which corresponds to 45% of all ongoing sick leave registrations [8].

The prevalence of work-related stress in Sweden has been reported to be 38%, which is amongst the highest levels in Europe [10]. Exhaustion disorder in a Swedish working population has been estimated at 13% [11]. While women who work in healthcare and other people-oriented human services professions are affected to a greater extent [11, 12], a substantial increase amongst men has recently been noted [9]. An association to work demands and degree of work control has been shown in both genders [11].

Rehabilitation has been shown to take longer time with longer symptom duration before receiving a stress-related diagnosis [13]. Of working age patients seeking primary care, 59% have been reported to experience stress-related symptoms and of those, 9% met the criteria for exhaustion disorder regardless of consulting complaint [14]. Prolonged exposure to stress without opportunity for recovery may entail a gradual increase in number of symptoms, causing patients to seek care repetitively with the symptom being the presenting complaint [1]. Many consult a general practitioner (GP) long before their symptoms are severe enough to require sick leave [15]. It is also common that patients seek emergency or drop-in care and that they are examined and treated for the presenting complaint alone, with neither the patient nor the physician identifying symptoms as signs of stress that may develop into exhaustion disorder [1].

Somatic symptoms that frequently constitute the presenting complaint at the occasion when the exhaustion disorder is diagnosed, are: nausea, irritable bowl, headache, dizziness, palpitations, chest pain, back pain, musculoskeletal pain, abdominal pain, and feeling faint [16, 17]. Multiple somatic symptoms have been associated with mental disorders [18], as well as a risk for subsequent long spells of sickness absence [19]. No study has been found that examines which stress-related complaints patients with exhaustion disorder consult for in the years preceding their diagnosis. Early identification of patients at risk is critical in order to prevent the development of exhaustion disorder, and can potentially prevent or reduce sick leave and hence the burden on both patient and society. It is therefore important to explore presenting complaints during the years preceding receiving the diagnosis, to identify which symptoms precede exhaustion disorder.

The primary aim of this study was to investigate the frequency of different presenting complaints that may be related to stress in patients with a diagnosed exhaustion disorder, in the two years preceding receipt of the diagnosis. Secondary aims were to explore potential associations between stress-related presenting complaints and demographic factors, as well as comorbidity and other potentially stress-inducing factors.

## Methods

### Study design, setting and participants

This was a retrospective chart review, in which data from medical charts were scrutinized [20]. The study was conducted at a suburban healthcare centre in western Sweden. In March 2016, 8521 patients were registered at the centre. Eight GPs were employed. Charts from all adult patients in whom exhaustion disorder (code F438A) was diagnosed at the healthcare centre from January 2015 to August 2016, and for whom there were medical records from the two preceding years, were included in the study.

### Data collection and analysis

Medical charts on the included patients were reviewed for the two years preceding the date of the diagnosis. All presenting complaints that were potentially related to stress were extracted. The choice of these symptoms was based on earlier studies describing stress symptoms presented when receiving an exhaustion disorder diagnosis [13, 16, 17].

The primary outcome was the frequency of stress-related complaints for which the patient had consulted during the two years preceding the exhaustion disorder diagnosis. Data on demographic factors (gender, age, occupation), number of GP visits with stress-related complaints, and comorbidity with other mental and/or somatic diagnoses were also collected. The variables were collected into a case report form, which was developed, pilot-tested on 20 charts, and slightly revised.

The extracted data were entered into Excel, checked for accuracy against the case report forms, and then analysed in IBM SPSS Statistics, version 22.0. Age was categorised into the groups < 30 years, 31–40 years, 41–50 years, 51–60 years, and > 60 years. For some variables where there were too few patients in some of the age categories, the categories were further collapsed into two groups (< 40 years and 40+ years). Differences in presenting complaints and comorbidity between men and women, age groups, and occupation categories were analysed with Pearson’s chi-square. Significance was set at the 0.05 level.

### Ethical considerations

Ethics approval and participant consent was not necessary as it is not required for this type of retrospective medical chart review, according to the Swedish Ethical Review Act (SFS 2003:460). Written permission for the medical chart review was obtained by the manager of the healthcare centre and the project was registered in accordance with the Swedish Personal Data Act (SFS 1998:204) before data collection began. Only the first author had access to and reviewed the medical charts and no names or personal identification numbers were extracted. It is not possible to identify whose charts were included in the study.

## Results

**Fig. 1:**
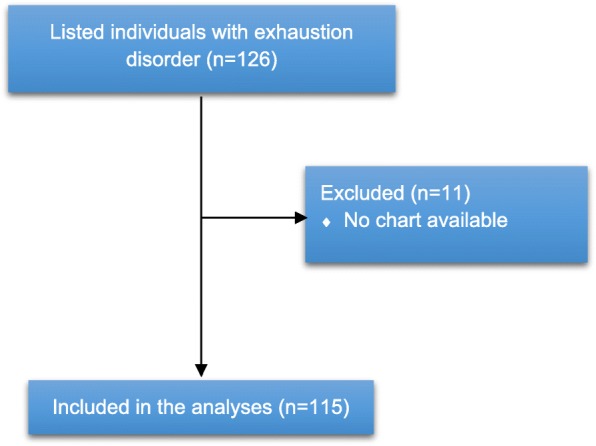
Flow chart of included patients

Between January 1, 2015 and August 31, 2016, exhaustion disorder was diagnosed in 126 patients at the healthcare centre. Of those, 11 patients had transferred from other healthcare centres and were excluded due to the unavailability of a medical chart for the two years preceding their receiving the diagnosis. Charts for 115 patients were included and reviewed (Fig. 1).

Of the 115 patients included in the study, 88 were women and 27 were men. Age ranged between 20 and 72 years, mean 47.1 (SD 10.8) years. Most patients with exhaustion disorder were found in the age group 41–50 years. Participant characteristics are presented in Table 2.

**Table 2:** Participant characteristics and data regarding number of GP visits and comorbidity (*n* = 115)

Variable	n (%)
Gender	
Women	88 (76)
Men	27 (24)
Age (years), mean (SD)	47.1 (10.8)
< 30 years	8 (7)
31–40 years	26 (23)
41–50 years	40 (35)
51–60 years	25 (22)
> 60 years	16 (14)
Occupation	
Office work	37 (32)
Industrial work^a^	7 (6)
Education	24 (21)
Health care	19 (16)
Other work	17 (15)
Unemployed	9 (7.8)
Student	2 (2)
Comorbidity, somatic diagnoses	70 (61)
Comorbidity, mental diagnoses	61 (53)
Number of GP visits, mean (SD)	5.2 (3.7)
Number of GP visits, median (range)	4.0 (0–18)

^a^Industrial work includes blue collar professions, e.g. factory workers, carpenters

The number of symptoms for which patients had consulted during the two years preceding diagnosis ranged from 0 to 12, with a mean of 3.7 (SD 2.2). A majority, 89.6%, had consulted for 6 symptoms or less. There were no gender or age differences in the number of presenting symptoms. Frequencies of the different presenting complaints in the two years preceding diagnosis are presented in Table 2. The two most frequent complaints, for both women and men, were infections and anxiety/depression. Although the complaint pattern differed somewhat between men and women, none of the gender differences were significant.

For the majority of the presenting complaints, most patients were in the age group 41–50 years. Few patients younger than 30 years had consulted a GP before receiving their diagnosis. The only complaints registered for this age category were gastrointestinal symptoms, anxiety/depression and infection. Chest pain complaints were evenly distributed across the age categories. Fatigue was distributed across all age categories, although the largest proportion, 100%, was found in patients older than 60 years. When age was categorised in two groups, a significant difference between the age groups were seen for two of the presenting complaints. The proportion of patients older than 40 years who consulted for back pain (17% vs. 3%, *p* = 0.037) and fatigue (42% vs. 18%, *p* = 0.012), respectively, was significantly higher than in those younger than 40 (Table 3).

**Table 3:** Stress-related presenting complaints during the two years preceding the receipt of the diagnosis exhaustion disorder, by gender and age group (*n* = 115)

Complaint	Total (n = 115) n (%)	Women (*n* = 88) n (%)	Men (*n* = 27) n (%)	*p*-value	< 40 years (*n* = 34) n (%)	40+ years (*n* = 81) n (%)	*p*-value
Infection	56 (49)	44 (50)	12 (44)	0.613	16 (47)	40 (49)	0.820
Anxiety/depression	53 (46)	39 (44)	14 (52)	0.492	14 (41)	39 (48)	0.494
Stress	45 (39)	36 (41)	9 (33)	0.490	11 (32)	34 (42)	0.335
Other pain	41 (36)	34 (39)	7 (26)	0.228	9 (26)	32 (40)	0.183
Fatigue	40 (35)	33 (38)	7 (26)	0.269	**6 (18)**	**34 (42)**	**0.012**
Gastrointestinal symptoms	38 (33)	29 (33)	9 (33)	0.971	11 (32)	27 (33)	0.919
Sleep disturbance	36 (31)	27 (31)	9 (33)	0.795	10 (29)	26 (32)	0.777
Headache	17 (15)	15 (17)	2 (7)	0.217	4 (12)	13 (16)	0.555
Dizziness	17 (15)	15 (17)	2 (7)	0.217	5 (15)	12 (15)	0.988
Chest pain	16 (14)	12 (13)	4 (15)	0.877	6 (18)	10 (12)	0.453
Back pain	15 (13)	14 (16)	1 (4)	0.099	**1 (3)**	**14 (17)**	**0.037**
High blood pressure	13 (11)	10 (11)	3 (11)	0.971	1 (3)	12 (15)	0.067
Palpitations	9 (8)	8 (9)	1 (4)	0.362	1 (3)	8 (10)	0.206
Other complaints	24 (21)	21 (24)	3 (11)	0.154	7 (21)	17 (21)	0.962

Differences between genders and age groups were analysed using Chi square. Bold numbers indicate a significant difference

Infection, stress, anxiety/depression, and gastrointestinal symptoms were frequent presenting complaints across all occupational categories. Regardless of occupational category, the patients had consulted several times for these symptoms before receiving their diagnosis. The most frequent complaint the years before diagnosis in office workers were infection (reported by 59%), and in industrial workers, anxiety/depression (71%). In school employees, the most frequent complaint was anxiety/depression (63%), and in healthcare workers, stress (42%) (Table 4). Differences between occupational categories could not be analyzed due to too small numbers in some of the categories.

**Table 4:** Stress-related presenting complaints during the two years preceding the receipt of the diagnosis exhaustion disorder, by occupation (*n* = 115)

Complaint	Office (*n* = 37) n (%)	Education (*n* = 24) n (%)	Health care (*n* = 19) n (%)	Industry (*n* = 7) n (%)	Other occupation (*n* = 17) n (%)	Student (n = 2) n (%)	Unem-ployed (*n* = 9) n (%)
Infection	22 (60)	11 (46)	6 (32)	3 (43)	7 (41)	2 (100)	5 (56)
Anxiety/depression	10 (27)	15 (62)	7 (37)	5 (71)	11 (65)	1 (50)	4 (44)
Stress	15 (40)	12 (50)	8 (42)	4 (57)	2 (12)	1 (50)	3 (33)
Other pain	13 (35)	12 (50)	6 (32)	1 (14)	4 (24)	–	5 (56)
Fatigue	10 (27)	11 (46)	4 (21)	2 (29)	10 (59)	–	3 (33)
Gastrointestinal symptoms	12 (32)	9 (38)	5 (26)	4 (57)	4 (24)	1 (50)	3 (33)
Sleep disturbance	11 (30)	10 (42)	5 (26)	2 (29)	6 (35)	–	2 (22)
Headache	3 (8)	5 (21)	5 (26)	1 (14)	3 (18)	–	–
Dizziness	3 (8)	5 (21)	5 (26)	–	4 (24)	–	–
Chest pain	6 (16)	3 (12)	1 (5)	–	4 (24)	–	2 (22)
Back pain	3 (8)	5 (21)	2 (10)	1 (14)	4 (24)	–	–
High blood pressure	3 (8)	4 (17)	2 (10)	1 (14)	3 (18)	–	–
Palpitations	2 (5)	4 (17)	1 (5)	–	2 (12)	–	–
Other complaints	5 (14)	9 (38)	3 (16)	–	4 (24)	–	3 (33)

With regard to comorbidity, 53% of the patients (50% of women, 63% of men) had another present or prior other mental diagnosis, for instance general anxiety disorder or depression. Somatic comorbidity was present in 61% (62% of women, 56% of men), and included primarily hypothyroidism, hypertension, diabetes, and cardiovascular disease. The proportion of patients with mental or somatic comorbidity was significantly higher in patients older than 60 years (*p* = 0.029 and *p* <  0.001, respectively) (Table 5).

**Table 5:** Mental and somatic comorbidity, by age group and occupational category (*n* = 115)

	Other mental diagnosis (*n* = 61) n (%)	Somatic diagnosis (*n* = 70) n (%)
Age group		
< 30 years	2 (25)	3 (38)
31–40 years	13 (50)	6 (23)
41–50 years	19 (48)	28 (70)
51–60 years	13 (52)	17 (68)
> 60 years	**14 (88)**	**16 (100)**
p-value	0.029	< 0.001
Occupation		
Office workers	23 (62)	22 (60)
Industry	4 (57)	4 (57)
Education	12 (50)	17 (71)
Health care	7 (37)	11 (58)
Other occupation	7 (41)	10 (59)
Student	0 (0)	0 (0)
Unemployed	**8 (89)**	6 (67)
p-value	0.025	0.711

Bold numbers indicate a significant difference. Differences between age groups and occupational categories were analysed using Chi square

Comorbidity with other mental diagnoses in different occupational categories varied from 37% in healthcare workers to 62% in office workers. Comorbidity with somatic diagnoses ranged between 57 and 71%. No comorbidity were seen in students. The proportion of mental comorbidity was significantly higher in the unemployed patients (*p* = 0.025), while no significant differences were found in somatic comorbidity (Table 5).

Potentially stress-inducing factors were identified in 35 (30%) of the patients. These were factors that indicated a stressful situation not only in the workplace but also at home, and included financial situation, alcohol abuse, and family illness. Twenty-five of the patients (22%) had a family member, most frequently a child, with a mental, neuropsychiatric or somatic disease. Several patients had family members who also suffered from exhaustion disorder.

## Discussion

This medical chart review of patients with exhaustion disorder showed that the disease was more prevalent in women than in men at this healthcare centre in western Sweden, and that individuals between 40 and 50 years of age were the most affected. The patients sought care for many different reasons in the years preceding their diagnosis; the two most common complaints were infection and anxiety/depression, presented by approximately half of the patients. Other stress-related complaints that were seen to a large degree were stress, other pain, fatigue, gastrointestinal symptoms, and sleep disturbance. There were no gender differences, and the only age differences were that fatigue, back pain, and comorbidity were more frequent in older patients. More than half of the patients had comorbidity with other mental or somatic diseases, and comorbidity was also linked to higher age.

The wide variety of symptoms for which the patients consulted their GP during the years before their diagnosis is consistent with previous research showing how the body reacts to sustained periods of stress. Several studies have shown that the development of many different physical and mental symptoms of stress can be part of developing an exhaustion disorder [2, 9, 21].

The two most common presenting complaints were infection and anxiety/ depression. High susceptibility to infection has earlier been linked to longstanding stress [16], suggesting that a red flag should be raised when patients consult with complaints of infection. Although symptoms of anxiety or depression could indicate anxiety disorder or depression, they may also be a sign of stress and part of developing exhaustion disorder. This implies that a particularly thorough patient history should be taken for patients presenting with these complaints, to explore potential high exposure to stress factors.

Other frequent complaints were stress, pain, fatigue, gastrointestinal symptoms, and sleep disturbances. These are common presenting complaints in general practice and should attract the physician’s attention and be cause for further exploration of potential stress-related causes of the symptoms [16–18]. Sleep disturbance, anxiety, fatigue and back pain are symptoms that have increased steadily among working individuals since the 1980s [9].

Although the complaint pattern differed slightly between men and women, we did not find any significant differences. This finding is consistent with earlier research, in which no significant gender differences in neither mental or somatic symptoms were found in patients with exhaustion disorder [13, 17].

Exhaustion disorder was most frequent in the age group 41–50 years. A potential reason for this could be the accumulation of stressors from both work and family life, which creates multiple demands on the individual to balance work and life priorities. The finding is supported by a Danish study that found a higher prevalence of mental disorder in patients older than 40 years than in younger patients [22]. Patients in this age group also had the most number of GP visits in the years preceding diagnosis, underscoring the importance of paying close attention to this age group. Two symptoms, back pain and fatigue, were more frequent among older patients. This is in line with earlier research that has shown that musculoskeletal pain is more frequent in older patients with exhaustion disorder [17]. Although back pain and other musculoskeletal pain is prevalent in older patients in general, our findings suggest that these symptoms may be related to exhaustion disorder and that GPs should be aware of this association.

Most of the patients with exhaustion disorder were office workers, in whom high work demands and often limited work control are common [16]. Many were employed in education and health care. Earlier studies have shown differences in the development of exhaustion disorder amongst individuals in different types of work in combination with socioeconomic factors [12]. The development of exhaustion disorder has been shown to be associated with stress in the workplace and with low socioeconomic status [11]. Women with exhaustion disorder often work in health care, social services and education [12]. In the present study, sleep disturbances, back pain, other pain, palpitations, high blood pressure, gastrointestinal symptoms, anxiety/depression, stress, infection and fatigue were the most frequent complaints in patients who work in education. Education, as well as health care and social services, are areas that have been particularly exposed to policy changes, reorganization, and budget cuts in Sweden in recent years, which likely is a contributing factor to sustained work-related stress leading to exhaustion disorder.

The high degree of comorbidity with other mental disease or somatic disease seen in the present study, is also consistent with other studies. Exhaustion disorder is frequently seen together with other mental disorders, particularly depression and anxiety [23]. A high co-occurrence between most mental disorders for which patients consult primary care has been shown in a Danish population [22]. That study showed the highest comorbidity for anxiety disorders, where almost 90% also had another mental diagnosis. It is important that the patients receive the correct diagnosis since treatment to some extent differs [4, 8]. The Swedish diagnostic criteria specify that if anxiety, depression or dysthymia is present concurrently, that condition should be treated first and exhaustion disorder second [1]. Many patients in the present study had earlier been affected by exhaustion disorder, and the risk for recurrence appears to be high [5]. Even after recovery from exhaustion disorder, patients can have a high susceptibility for stress and are vulnerable to falling sick again.

It was notable from the chart reviews that stress was not solely related to work conditions. Many patients also had personal problems and other stressful situations, which has been shown to contribute to the development of exhaustion disorder [11]. An interesting finding was that several of the patients with exhaustion disorder had family members who also had been diagnosed with the same disease, most often a spouse or adult child. Many had children with a mental, neuropsychiatric or somatic diagnosis, which can be extremely stressful for the parent. To prevent disease it is therefore important that the doctor and other healthcare professionals take the time to listen to their patients and include both work and personal/family factors in their assessment. It is likely that a combination and interaction of work-related, environmental and personal factors may lead to aggravated symptoms and a more pronounced exhaustion disorder.

### Study limitations

This was a retrospective observational study, without control group, which means that no firm conclusions can be drawn regarding possible associations between prior symptoms and receiving the diagnosis of exhaustion disorder. The retrospective medical chart review method has both limitations and strengths. Potential limitations, in addition to the retrospective design, are incomplete documentation and the possibility of missing records, as well as variance in the quality of information recorded in the charts. Advantages include that the method makes it possible to use readily available existing data that is clinically relevant, and that the results can be used to generate hypotheses that can be tested prospectively in future research [24].

The presenting complaints that were registered are complaints which may be associated with stress and exhaustion, as confirmed by earlier research [16–18], but could also be associated to a somatic disease or to other mental disease, and we cannot be certain that they were linked to stress. Some of the symptoms, e.g. chest pain or gastrointestinal symptoms might be due to other (somatic) pathology, which was not analysed in our study. Furthermore, infection and anxiety/depression were the two most prevalent presenting complaints, and it must be kept in mind that these are common diagnoses and only a small proportion of these complaints might be related to exhaustion.

Other limitations are that only one author reviewed the charts, which might have led to omissions and errors; and that only charts from the healthcare centre in which the patients were registered were reviewed, which means that presenting complaints for which the patients had consulted elsewhere, e.g. emergency departments, were not considered. The healthcare centre from which the data were collected is located in an area where the population is homogenous (with few immigrants) and has a higher than average socioeconomic status, which may reduce generalisability of the findings to other, more heterogeneous, contexts. Only GP visits were extracted from the medical chart. Telephone contact with both doctors and nurses for possible stress-related complaints were not registered.

### Implications for practice and research

Infection and anxiety/depression, the two most prevalent presenting complaints, are very common complaints in primary care, seen by GPs on a daily basis. In view of the high prevalence of these complaints in patients who later develop exhaustion disorder, it may be of relevance for GPs to pay extra attention to these complaints, allow sufficient time for an extended patient history, and explore whether the patient is exposed to stress in work or personal life. An example of how GPs could better address the possibility of symptoms being related to exhaustion disorder, would be to pay extra attention with recurring or long-lasting infections that may signal a dysfunctional immune system. Another example could be to allow extra time for an in-depth patient history and screen for stress exposure by exploring potential work-related and personal stressors, in patients who consult for the symptoms identified in this study.

Screening for stress exposure can be done with a screening instrument and increased use of such an instrument may help the GP to identify exhaustion disorder. Examples of screening instruments are the Self-reported exhaustion disorder Scale (s-UMS) [25] and the Karolinska Exhaustion Disorder Scale (KEDS) [26]. To address work-related stress more specifically, the Work Stress Questionnaire (WSQ) could be used [27]. Any of these instruments would be a good supplement to the clinical assessment that may help the GP to reach a correct diagnosis.

The wide variety of presenting complaints preceding the exhaustion disorder diagnosis confirms the clinical perception that this condition is challenging to manage due to large individual variations in characteristics and symptoms. Typical management is based on regular follow-ups and may include: identification of work-related and personal stressors, counselling (including recommendations for physical activity, sleeping and eating habits, coping strategies and stress management), referral for psychological counseling, physiotherapy or occupational therapy, issuing full or partial sick leave, and collaboration with employers. The patients had consulted their GP an average of 5 times for stress-related complaints during the two years preceding their diagnosis. This consulting frequency suggests that GPs meet these patients with stress-related symptoms well before their exhaustion disorder is diagnosed, providing ample opportunities for early identification of these patients. Early identification of stress-related symptoms, both work-related and personal, could allow the GP to discuss and suggest suitable preventive measures, which might lower the risk for future sick leave [28].

The identified presenting complaints, particularly infections and anxiety/ depression, are symptoms that GPs may need to be concerned about, and that may allow them to earlier identify these symptoms as potentially stress-related and to take measures to prevent the development of a manifest exhaustion disorder. Because exhaustion disorder was most frequent in individuals between 41 and 50 years, and these patients also had a high frequency of visits in the years preceding diagnosis, GPs should pay particular attention to this age group and explore possible stress-related factors in greater depth.

Future research is needed to examine whether the results of this study are reproducible in other geographical areas with different socioeconomic conditions, in Sweden as well as in other countries. It would also be relevant to explore presenting complaints and risk factors by reviewing medical charts from hospital visits, including emergency units, to gain knowledge about patients’ care-seeking patterns at different care levels for their stress-related symptoms. Potential associations between stress-related presenting complaints and exhaustion disorder also needs to be explored using a prospective study design.

## Conclusions

This study shows that patients with exhaustion disorder may have consulted their GP numerous times with stress-related complaints in the years preceding their diagnosis. The most common complaints for which patients consulted a GP in the years preceding diagnosis were infection and anxiety/depression. Other frequent complaints were stress, other pain, fatigue, gastrointestinal symptoms, and sleep disturbances. Comorbidity with other mental or somatic diseases was frequent.

The findings indicate which presenting complaints GPs may need to pay extra attention to, as well as in which age category and professions most patients develop exhaustion disorder. The study provides new knowledge that may facilitate identification of these patients at an early stage so that necessary help and support can be provided earlier. Increased awareness of patients who already have another mental disease and/or a somatic disease is also important, since the study showed a high prevalence of comorbidity. Addressing stress factors earlier in the course of illness and preventing the development of exhaustion disorder may contribute to a reduced burden for both the individual patient and for the society, with a reduction in sick leave and societal costs for mental illness.

## Abbreviations

GP: General practitioner
SD: Standard deviation
